# Intradural Intramedullary Mixed Type Hemangioma: Optimizing the Surgical Management through Intraoperative Neurophysiological Monitoring

**DOI:** 10.1155/2015/984982

**Published:** 2015-12-29

**Authors:** Ahmad Jabir Rahyussalim, Adrian Situmeang, Ahmad Yanuar Safri, Zulfa Indah K. Fadhly

**Affiliations:** ^1^Department of Orthopaedic and Traumatology, Faculty of Medicine, University of Indonesia, Jakarta 10430, Indonesia; ^2^Neurophysiology Division, Department of Neurology, University of Indonesia, Jakarta 10430, Indonesia

## Abstract

Intradural intramedullary mixed type hemangioma is a rare histotype of primary spinal cord tumors, though it can carry a severe clinical burden leading to limb dysfunction or motor and sensory disturbances. Timely intervention with radical resection is the hallmark of treatment but achieving it is not an easy task even for experienced neurosurgeons. We herein present an exemplificative case presenting with sudden paraplegia in which total resection was achieved under intraoperative neurophysiology monitoring. A thorough discussion on the operative technique and the role of neuromonitoring in allowing a safe surgical management of primary spinal cord tumors is presented.

## 1. Introduction

Primary spinal cord tumors are uncommon but associated with high morbidity [1]. They account for 2% to 4% of all central nervous system neoplasms with an incidence of 2.5–8.5 per 100.000 people per year [1, 2]. Among them, intramedullary lesions represent 0.3% of all spinal cord tumors [3]. Patient typically presents with pain, numbness, weakness, and loss of limb coordination as well as bladder and bowel problems. The clinical presentation may be similar to intra- and extraspinal pathologies, including nonneoplastic lesions. Similar to those nonneoplastic causes, also primary spinal cord tumors are generally benign in nature due to their slow growing; for this they are usually classified as low grade (grades I and II) according to the World Health Organization (WHO) pathology classification [1, 4, 5]. Of all the rare tumors presenting in the spinal cord, hemangiomas are the third most common ones. Being congenital vascular malformations rather than true neoplasms, hemangiomas consist of vessels similar to those of embryonic capillaries (grade I WHO) [6]. These relatively benign lesions grow very slowly, reaching large sizes, and may lead to compression of the spinal cord causing delayed and/or sudden symptoms.

Hemangiomas can be classified histologically into 3 types: capillary, cavernous, and mixed-type. Capillary hemangiomas contain capillaries which are normal in size but with sparse fibrous stromal, whereas cavernous hemangiomas contain enlarged blood vessels lined by flat endothelium [6]. In less than 30% of cases, hemangiomas can be found as intradural extramedullary lesions if they arise from the spinal nerve roots [7]. Complete surgical resection remains the primary treatment for patients with this neoplasm. Neurological impairment may be caused by their space occupying effect, as well as chronic or acute bleeding. Most extradural and intradural extramedullary tumors can be surgically resected while most intramedullary tumors are infiltrative in nature and complete resection is difficult. Surgery also carries risk of worsening neurological deficits and death due to damage to the motor or sensory tract [8]. Lower complication rate was found for patient who had surgery with intraoperative neurophysiologic monitoring (IOM) [9]. In this case report, an exemplificative case of intradural intramedullary mixed-type hemangioma treated at Cipto Mangunkusumo Hospital is presented and the surgical management of the condition is discussed.

## 2. Case Illustration

A previously healthy 35-year-old male presented with short history of pain, gradual numbness in both the lower limbs, and bladder and bowel retention followed by complete paralysis. Patient had dull, aching, nonradiating pain, starting 20 days prior to admission to our hospital. Simultaneously, he had gradual numbness and develops problems of urinating and defecating. Within a period of a week, there was paraplegia with grade 0 power of both his lower limbs except for plantar-flexion of the ankle joint which is grade 1 power. Complete sensory deficit below T10 occurred along with sphincter disturbances characterized by urinary and bowel retention requiring catheterization. Muscles were hypertonic with exaggerated jerk reflex and clonus.

**Figure 1:**
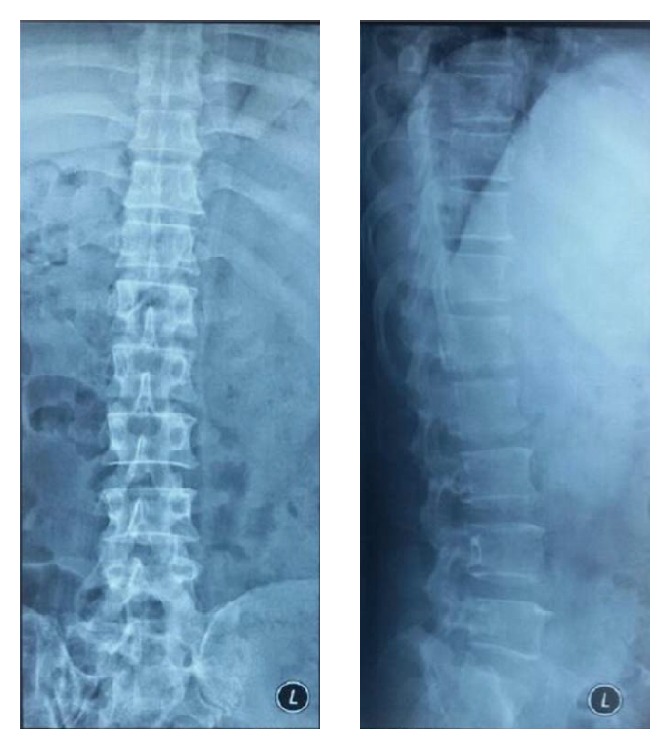
Initial AP and lateral view X-rays showing only slight loss of lumbar lordosis (taken on 20 May 2015).

**Figure 2:**
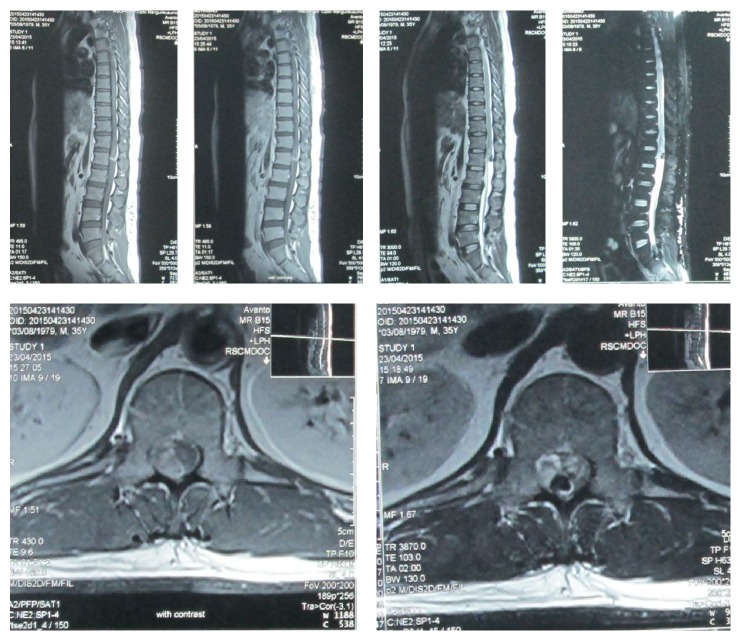
Sagittal and axial view of T1 and T1 with contrast-weighted images showing intradural intramedullary hyperintense signal at L1 level of spinal cord. Sagittal and axial T2-weighted images showing isointense signal of the lesion (taken on 23 June 2015).

**Figure 3:**
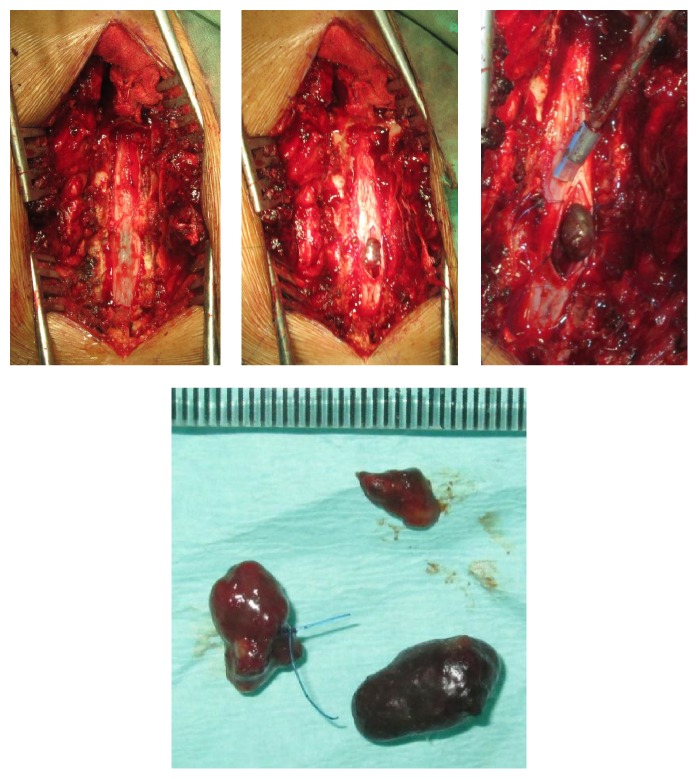
Surgical exposure and gross pathology of the tumor showing well-defined tubular lesion with dark-red color.

**Figure 4:**
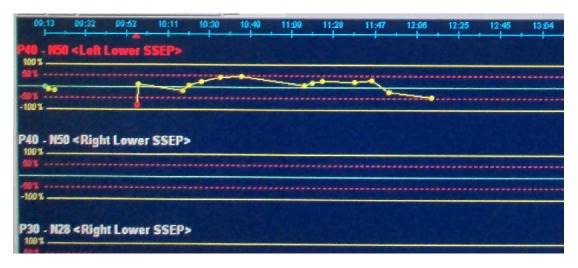
Deterioration of SSEP monitored at 11:47–12:00 PM.

**Figure 5:**
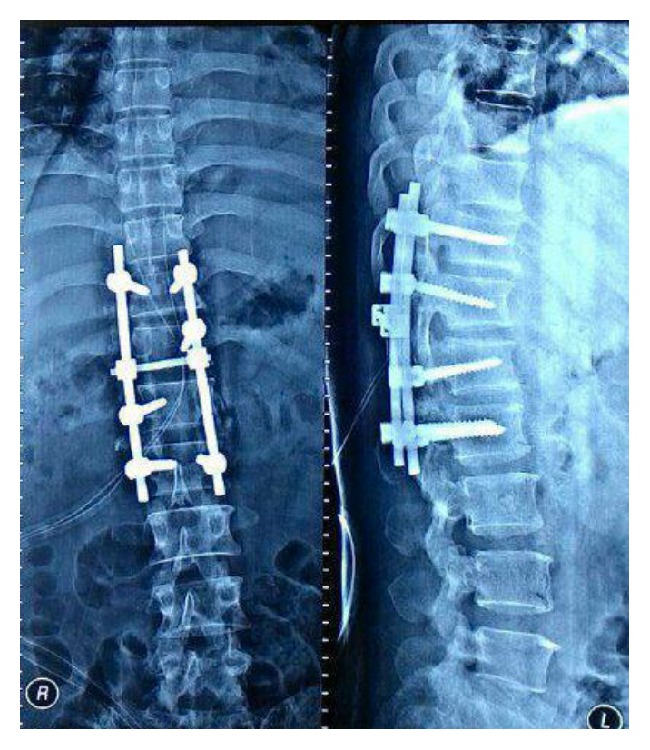
AP and lateral post-op X-rays of the patient showing posterior stabilization with pedicle screw and rod system and posterolateral fusion (taken on 21 June 2015).

Chest and thoracolumbar X-rays were taken and the result was normal with only slight loss of lumbar lordosis (Figure 1). T1-weighted magnetic resonance imaging showed irregular, hyperintense intramedullary lesion behind the body of L1 vertebrae predominantly on the right side. The lesion displaced the cord to the left. On T2-weighted magnetic resonance imaging, the lesion was isointense relative to normal cord. Gadolinium contrast administration revealed only slight enhancement of its surrounding wall (Figure 2). Surgery was performed under general anesthesia and continuous neurophysiology monitoring including MEP (motor evoked potentials), SSEP (somatosensory evoked potentials), and free-running EMG (electromyography). A posterior approach to the lumbar spine was performed with total laminectomy at T12-L1 level and partial laminectomy at T11 and L2 level. The posterior facet joints of T11 and L2 were preserved bilaterally. Following the laminectomy stage, the thecal sac was incised longitudinally revealing the tumor between the cords of cauda equina. Swelling and inflammation of the spinal cord were due to a well-defined tubular tumor with dark-red in color sized 3 × 1 × 0.5 cm (Figure 3). Complete resection of the tumor was performed with only slight deteriorations of the SSEP (Figure 4). At the end of the procedure, posterior stabilization with pedicle screw and rod system with posterolateral fusion was performed above and below the laminectomy level (Figure 5). Under hematoxylin-eosin staining, the tumor showed proliferation of blood vessels with variant size (capillary and cavernous-type). The vessels were lined with endothelium and filled with erythrocytes. The final diagnosis was mixed type intradural intramedullary spinal cord hemangioma. Postoperatively, the patient's spasticity in both lower limbs decreased but no restoration of movement was noted in the joints. Given the low-grade nature of the hemangioma, neither adjuvant radiotherapy nor chemotherapy was advised.

## 3. Discussions

Due to its rarity, tumors arising from the spinal cord can be difficult to diagnose. According to anatomic location, spinal cord tumors can be classified into 3 categories [1]: extradural, intradural extramedullary, and intradural intramedullary. Each of these categories has its own differential diagnosis that aid in the process of establishing diagnosis. Most extradural tumors consist of metastases even though some cases of malignant or benign tumor had been reported [10, 11]. The majority of intramedullary spinal cord tumors are gliomas which are mostly ependymomas and astrocytomas. The third most common one is hemangioma or hemangioblastoma, representing approximately 3% to 8% of all [1]. There is association between intradural hemangioma with von Hippel-Lindau (VHL) syndrome, an autosomal-dominant chromosomal disorder caused by deletion of chromosome 3p, especially if the tumor is multiple [12]. Meningiomas or peripheral nerve sheath tumors comprise most of intradural extramedullary spinal cord tumors [1, 3]. The clinical presentation of spinal cord tumors is determined by anatomic location of the tumor, size, and the nature of the tumors itself. Pain is the most common chief complaint and may present as back pain, radicular pain, or central pain. Motor disturbance followed by sensory loss is the next most common complaint. Least of all, sphincter malfunction such as bowel and bladder retention is seen only in small number of patients [13]. Almost every spinal cord tumors present with signs and symptoms mentioned above and the use of advanced imaging such as MRI is compulsory.

Spinal hemangiomas are congenital vascular malformations which affect males and females equally [6]. The peak presenting age is the fourth decade of life with chronic progressive signs and symptoms mentioned above. Acute worsening is due to new bleeding source within or around the lesion [2, 6]. As WHO grade I capillary-rich neoplasms, hemangioma usually appears as well-circumscribed, nodular masses with variable T1 signal (mostly isointense), enhancement with gadolinium, and hyperintense on T2-weighted images [7]. Other common findings are flow voids, adjacent cysts, and hemorrhage. Contrary to that, the patient presented in this case has hyperintense signal on T1-weighted images and isointense signal on T2-weighted images. Upon gadolinium administration, the lesion also only partially enhanced. This is what makes the diagnosis of hemangioma unlikely before intraoperative gross pathology findings reveal tumor similar to the characteristic of hemangioma. As a gold standard for diagnosis, histopathological findings of hematoxylin-eosin-stained section showed findings consistent of hemangioma without any cellular atypia.

Treatment of this neoplasm consists of total excision under high magnification using microsurgical techniques for the amenable tumor and stereotactic radiosurgery for the unresectable one [1, 6, 14]. As in many other neurosurgical interventions on highly vascularized lesions, or involving major vessels, the removal of spinal hemangiomas is at high risk of intraoperative bleeding. The importance of accurate hemostasis should therefore be stressed particularly in those cases, and the use of advanced hemostatic should be encouraged [15]. Furthermore, the partial removal of the tumor should be avoided if possible as it might lead to prolonged postoperative bleeding, local recurrences, or postoperative radiological changes difficult to interpret [16, 17].

The goals of the surgical management must be a total excision without neurological morbidity: for this to be feasible, the use of neuromonitoring should always be kept in mind. Three modalities are considered of importance during resection of spinal cord tumors; those are MEP, SSEP, and free-running EMG. MEP are used to monitor the integrity of the anterior and lateral part of the spinal cord whereas SSEP are used to monitor the posterior part during surgical division of the tumor from the surrounding healthy cords. Free-running EMG is helpful in detecting nerve root irritation during instrumentation. Intraoperative neurophysiological derangements are mostly reversible through temporary intervention [9, 18]. Surgical delay, warm saline irrigation, and improving blood perfusion have proved useful for improvement [18]. Therefore, IONM is able not only to predict but also to prevent deterioration. In this opinion, it is best and should be considered as gold standard to combine surgery with IONM in spinal cord tumor surgery. Surgeons should be warned for the increased risk of adverse neurological outcomes. Some Neurophysiological waves changes in IONM such as decreased of SSEP amplitude could be happened [19].

## 4. Conclusions

Hemangioma of spinal cord should be considered in the differential diagnosis of patients with progressive neurological deterioration even though the radiological findings are inconsistent. Timely intervention with complete removal of symptomatic intradural intramedullary hemangioma is the hallmark of treatment; to achieve it, all adjuvant modalities including intraoperative neurophysiological monitoring should be considered to prevent postoperative deficits and predict the long term outcome. Adjuvant radiation therapy may be considered only for subtotal removal case or in cases not amenable for surgical resection.
